# NORE1A Regulates MDM2 Via β-TrCP

**DOI:** 10.3390/cancers8040039

**Published:** 2016-03-23

**Authors:** M. Lee Schmidt, Diego F. Calvisi, Geoffrey J. Clark

**Affiliations:** 1 Department of Pharmacology and Toxicology, James Graham Brown Cancer Center Molecular Targets Group, University of Louisville, Louisville, KY 40202, USA; lee.schmidt@louisville.edu; 2 Department of Clinical and Experimental Medicine, University of Sassari, Sassari 07100, Italy; calvisid@uniss.it

**Keywords:** Ras, NORE1A, MDM2, β-TrCP

## Abstract

Mouse Double Minute 2 Homolog (MDM2) is a key negative regulator of the master tumor suppressor p53. MDM2 regulates p53 on multiple levels, including acting as an ubiquitin ligase for the protein, thereby promoting its degradation by the proteasome. MDM2 is oncogenic and is frequently found to be over-expressed in human tumors, suggesting its dysregulation plays an important role in human cancers. We have recently found that the Ras effector and RASSF (Ras Association Domain Family) family member RASSF5/NORE1A enhances the levels of nuclear p53. We have also found that NORE1A (Novel Ras Effector 1A) binds the substrate recognition component of the SCF-ubiquitin ligase complex β-TrCP. Here, we now show that NORE1A regulates MDM2 protein levels by targeting it for ubiquitination by SCF-β-TrCP. We also show the suppression of NORE1A protein levels enhances MDM2 protein expression. Finally, we show that MDM2 can suppress the potent senescence phenotype induced by NORE1A over-expression. Thus, we identify a mechanism by which Ras/NORE1A can modulate p53 protein levels. As MDM2 has several important targets in addition to p53, this finding has broad implications for cancer biology in tumor cells that have lost expression of NORE1A due to promoter methylation.

## 1. Introduction

Mouse Double Minute 2 Homolog (MDM2) is an E3 ubiquitin ligase and serves as a key regulator of the p53 master tumor suppressor [1]. MDM2 can negatively regulate p53 protein levels by binding and ubiquitinating p53. This post-translational modification results in the nuclear export of p53 and subsequent degradation by the 26S proteasome [2]. In addition, MDM2 is shown to regulate p53 mediated gene transcription by binding and inhibiting its activity as well as suppressing expression of the p53 gene locus [1]. However, this situation is further complicated by the observation that p53 can stimulate MDM2 transcription. Thus, p53 and MDM2 exist in a feedback loop, resulting in a complex, and likely tissue specific, relationship [3]. The frequently observed over-expression of MDM2 in primary tumor cells correlates well with the loss of p53 expression [4] and suggests a central physiological role for MDM2 dysregulation in human disease.

Activation of the Ras oncoprotein by point mutation occurs frequently in human tumors [5]. Ras is also frequently found constitutively activated in the absence of structural mutations, due to the loss of its negative regulators such as NF1 (Neurofibromin 1) and DAB2IP (DAB2 Interacting Protein) [6,7]. Hyper-stimulation of Ras signaling pathways are known to be transforming; however, constitutively high levels of Ras activity can conversely induce Oncogene Induced Senescence (OIS) in cells and this is thought to serve as a major barrier to Ras driven transformation and tumorigenesis [8,9]. Ras induces OIS, in part, via activation of p53 [9,10]. However, Ras can also upregulate MDM2 via the Raf/MAPK pathway, which should act to suppress p53 levels [11]. Thus, the mechanisms underlying the connection between Ras and p53 are intricate and involve both positive and negative effects [12]. The net effect of Ras on p53 in any given cell will likely depend upon the relative balance of positive and negative signaling machinery. 

NORE1A (RASSF5) is a member of the RASSF family of Ras effector/tumor suppressors [13,14]. It can link Ras to the induction of apoptosis and senescence and is frequently down-regulated by epigenetic means in human tumor cells. NORE1A mediates its tumor suppressive effects, in part, via p53. NORE1A can complex with p53, altering specific post-translational modifications of p53 [15]. Furthermore, NORE1A also promotes enhanced levels of nuclear p53 protein [16], but the mechanism of NORE1A induced p53 stabilization remains unclear.

Recently, we have shown that NORE1A forms an endogenous complex with the Skip-Cullin-F-Box-ubiquitin ligase E3 substrate recognition component β-TrCP, and can scaffold it to specific targets for ubiquitination and proteolytic degradation [17]. As NORE1A has been reported to complex with MDM2 [18], and as β-TrCP can promote the degradation of MDM2 [19], we wondered if NORE1A might be acting on p53, in part, via the degradation of MDM2.

Here we show that NORE1A regulates MDM2 protein levels and that this effect is β-TrCP dependent. Moreover, we show that Ras enhances endogenous MDM2 expression unless it is co-transfected with NORE1A, whereupon it suppresses MDM2 levels. Finally, we show that MDM2 suppresses the ability of NORE1A to induce senescence in a mutant Ras tumor cell line. These results explain how NORE1A may regulate nuclear p53 protein levels and why MDM2 may be over-expressed in many tumors. As MDM2 can regulate other important proteins involved in the control of cellular homeostasis, such as the retinoblastoma protein [20], the results may have much broader implications.

## 2. Results

### 2.1. NORE1A Suppresses MDM2 Protein Expression via β-TrCP

**Figure 1:**
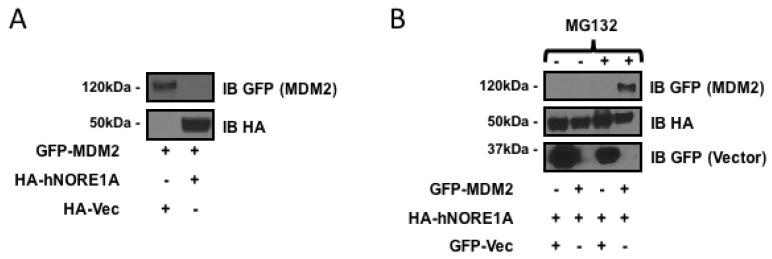
(**A**) HEK-293 cells transfected with expression constructs for MDM2 paired with an empty vector or a construct expressing NORE1A. MDM2 expression can be easily detected when co-expressed with an empty vector. Levels of MDM2 are suppressed when co-expressed with NORE1A; (**B**) HEK-293 cells were transfected with expression plasmids expressing NORE1A paired with either an empty vector or a construct expressing MDM2. 24 h post-transfection, the two groups were split and allowed to re-adhere to the culture dishes. Then the matched sets were treated with either DMSO (carrier) or MG132 at a final concentration of 10 μM for 12 h. The cells were lysed and examined on Western blot. Here we show that inhibition of the proteasome disrupts the ability of NORE1A to suppress MDM2, suggesting that indeed NORE1A is controlling MDM2 protein levels via the 26S proteasome. Representative blots of 2 independent experiments are shown in both panels.

NORE1A can target the SCF-β-TrCP ubiquitin ligase complex to specific substrates [17]. As MDM2 is a substrate of the complex and NORE1A can directly bind MDM2 [18], we measured the ability of NORE1A to suppress MDM2 expression. We co-transfected HEK-293 cells with GFP-tagged MDM2 or HA tagged NORE1A and after 48 h measured protein expression levels. Figure 1a shows that NORE1A effectively suppressed the expression of MDM2. To confirm that effects we had observed were due to proteosomal degradation of MDM2, we took HEK-293 cells again transfected with GFP-MDM2 paired with an empty vector or an HA-tagged NORE1A. 24 h post-transfection, the transfected cells were split into two groups, one treated with DMSO and the other treated with the proteasome inhibitor MG132. Figure 1b shows that MG132 suppressed the ability of NORE1A to inhibit MDM2 expression.

**Figure 2:**
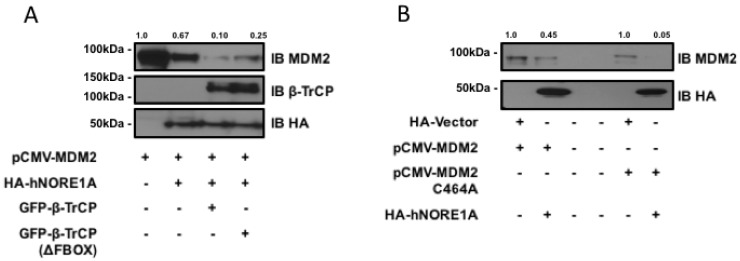
(**A**) HEK-293 cells were transfected with expression constructs expressing MDM2 paired with an empty vector or constructs expressing NORE1A, β-TrCP, or the dominant negative form of β-TrCP (ΔFBOX). NORE1A expression on its own has a substantial suppressive effect on MDM2 stability, and this effect is magnified when co-expressed with β-TrCP. However, the dominant negative β-TrCPΔFBOX reduces this effect, suggesting that NORE1A is cooperatively working with β-TrCP to regulate MDM2 protein levels; (**B**) MDM2 has the ability to self-ubiquitinate, and we sought to confirm whether NORE1A was degrading MDM2 via β-TrCP or inducing MDM2 auto-ubiquitination. We transfected HEK-293 cells with wild-type MDM2 and a mutant of MDM2 that is unable to auto-ubiquitinate (C464A) paired with an empty vector or NORE1A. We found that NORE1A was still able to suppress both the wild type and mutant MDM2, suggesting that indeed NORE1A is degrading MDM2 via β-TrCP, and not inducing MDM2 auto-degradation. Representative blots of 2 independent experiments are shown.

MDM2 can be ubiquitinated by β-TrCP and by an auto-ubiquitination activity. To determine if the degradation process was due to β-TrCP or the endogenous ubiquitin ligase activity of MDM2, we first repeated the experiment in the presence of a dominant negative mutant of β-TrCP. Figure 2a shows that the mutant inhibited the action of NORE1A on MDM2. The partial nature of the inhibition may be due to the presence of two isoforms of β-TrCP, only one of which is inhibited by the dominant negative. We then repeated the experiments on an MDM2 variant that has been mutated so that it loses intrinsic ubiquitin ligase activity [21]. Figure 2b shows NORE1A still suppressed the mutant MDM2 expression.

### 2.2. NORE1A Levels Determine if RAS Has a Negative or Positive Effect on MDM2

**Figure 3:**
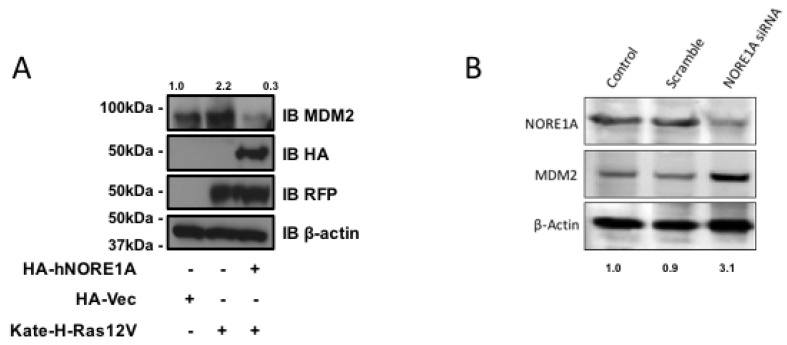
(**A**) HEK-293 cells were transfected with expression constructs expressing activated H-Ras paired with and without an expression construct for NORE1A. 24 h post-transfection the cells were lysed and levels of endogenous MDM2 were analyzed. When activated Ras was over-expressed, levels of MDM2 were found increased. However, when NORE1A was co-expressed with the activated Ras, this effect was reversed and the MDM2 levels were suppressed compared to the empty vector control; (**B**) HepG2 cells were transfected with a scramble siRNA control or a siRNA designed against NORE1A. 48 h post-transfection, the cells were lysed and the levels of endogenous MDM2 were analyzed. In the cells where NORE1A expression was suppressed, levels of MDM2 were observed to increase, again suggesting that indeed NORE1A is negatively regulating MDM2 protein levels. Relative intensities are represented in fold change to the control sample and representative blots of 2 independent experiments are shown.

NORE1A binds Ras directly and serves as a Ras effector. Therefore, it may serve as link between Ras and the regulation of MDM2. To determine if Ras is involved in the action of NORE1A on MDM2 we transfected Ras into HEK-293 cells in the presence or absence of NORE1A. In the absence of exogenous NORE1A, we found that Ras acted to enhance the levels of endogenous MDM2. However, when we included NORE1A in the Ras transfection, we found that the levels of MDM2 were suppressed (Figure 3a).

### 2.3. Inactivation of NORE1A Enhances MDM2 Expression

If NORE1A is truly regulating MDM2 expression, then we might expect that cells that have been suppressed for NORE1A should exhibit upregulated MDM2 protein levels. To determine if this is the case, we transiently transfected human liver tumor cells (HepG2) that retain NORE1A expression with a siRNA pool against NORE1A. Figure 3b shows that downregulation of NORE1A was clear by 48 h after transfection. The levels of MDM2 rose in the NORE1A suppressed cells.

### 2.4. MDM2 Suppresses NORE1A Mediated Senescence

**Figure 4:**
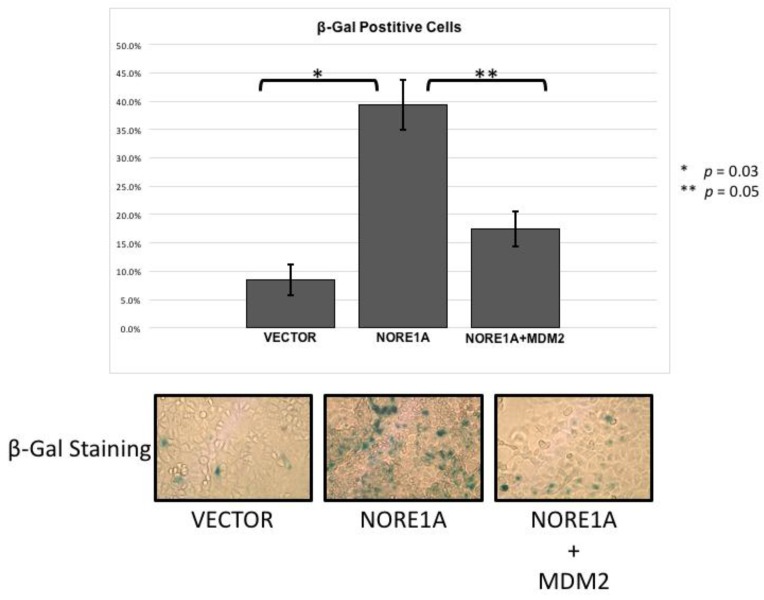
A549 cells were transfected with an empty vector, a construct expressing NORE1A, or NORE1A paired with 5x by mass of a construct expressing MDM2. In A549 cells expressing NORE1A, cells have exhibit a high level of senescence. This effect is suppressed in the presence of over-expressed MDM2. Representative pictures of β-gal staining from 2 independent assays are shown.

NORE1A is a potent Ras senescence effector that operates, in part at least, via p53 [15]. To determine if the suppression of MDM2 may play a role in the induction of senescence by NORE1A, we performed senescence assays in A549 lung tumor cells. These cells contain an activated Ras gene, wild type p53 but no NORE1A [15]. Transient transfections were performed with NORE1A in the presence or absence of MDM2. Figure 4 shows that the co-transfection of MDM2 could reduce the senescence induced by NORE1A. This suggests that elimination of MDM2 protein by NORE1A plays an important role in NORE1a and Ras mediated senescence.

## 3. Discussion

Aberrant activation of the Ras oncogene can lead to the development of Oncogene Induced Senescence (OIS). OIS suppresses the transforming effects of Ras and is a major barrier to tumor development [8,9]. The mechanisms underlying OIS and how it is subverted to facilitate tumor malignancy remain poorly understood.

NORE1A is a key Ras senescence effector that can promote both increased nuclear p53 levels and enhanced pro-senescent post-translational modifications of p53 [15,16]. NORE1A is frequently inactivated by an epigenetic process of promoter hyper-methylation in human tumor cells. In primary human tumor samples, the loss of NORE1A expression correlates with acquisition of enhanced malignancy and reduced pro-senescent signaling and activating post-translational modifications of p53 [15,16].

The level of p53 protein expression in a cell is controlled by many regulators, but one of the most important and best studied is the MDM2 protein. MDM2 acts at multiple levels to modulate p53 expression [3]. In particular, it ubiquitinates p53, promoting nuclear export and degradation by the 26S proteasome. As MDM2 suppresses p53, it is not surprising that it should exhibit oncogenic activity and show over-expression in many primary tumors [1,4]. In addition to p53, MDM2 has also been implicated in regulating a variety of other important mediators of cellular homeostasis, including the Rb tumor suppressor [20]. Therefore, the action of MDM2 in a cell is both powerful and multi-faceted.

**Figure 5:**
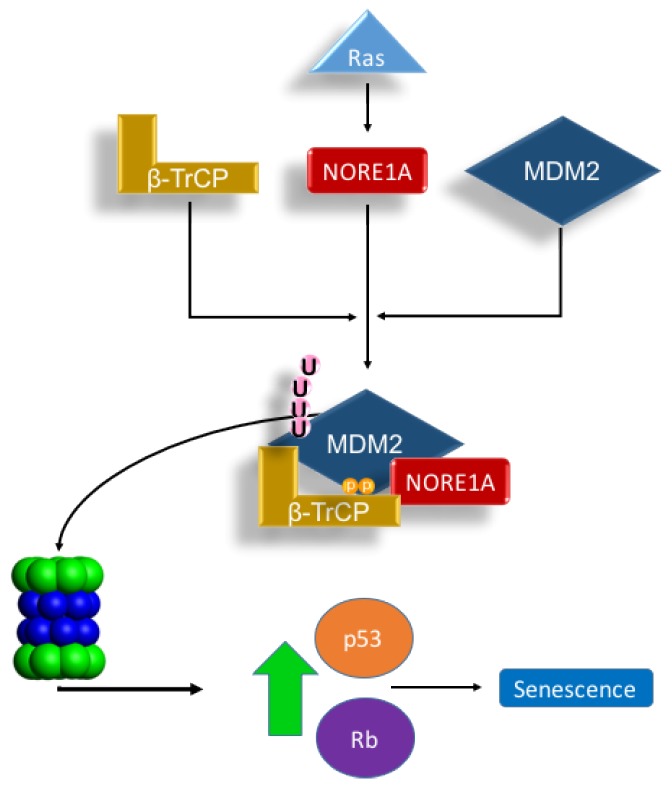
Scheme depicting our proposed Ras/NORE1A/β-TrCP/MDM2 senescence mechanism. The interaction of NORE1A to β-TrCP is Ras regulated, and this interaction regulates MDM2, a key negative regulator of p53 and Rb. Upon stimulation from Ras, MDM2 levels are suppressed by NORE1A and β-TrCP allowing levels of p53 and Rb to rise and activate an oncogene induced senescence mechanism. Accordingly, loss of NORE1A removes this key MDM2 regulatory mechanism, allowing for levels of MDM2 to increase resulting in excessive down-regulation of powerful tumor suppressive mechanisms operated by p53 and Rb.

MDM2 regulation is complex. At the protein level, this ubiquitin ligase is itself regulated by ubiquitin ligases such as SCF-β-TrCP [19]. Moreover, MDM2 also has an auto-ubiquitination function, which allows self-regulation [21]. We have observed that NORE1A not only appears to stabilize nuclear p53, but also binds directly to the β-TrCP complex [17]. NORE1A has recently been shown to bind MDM2 [18], but the potential for NORE1A to modulate MDM2 stability was not considered. We hypothesized that NORE1A might be acting, in part, to regulate p53 via MDM2 degradation. Our results show that NORE1A does indeed target MDM2 for proteosomal degradation. Moreover, by using an MDM2 mutant that cannot auto-ubiquitinate, as well as a dominant negative form of β-TrCP, we confirmed that the ubiquitination was due to β-TrCP and not MDM2 itself. As the binding of β-TrCP to NORE1A is Ras dependent [17], it is not surprising that we found NORE1A appears to couple Ras to the degradation of MDM2 (Figure 5).

RASSF1A is related to NORE1A and is better characterized as a tumor suppressor [13]. RASSF1A has also been reported to complex with MDM2 and promote its degradation to stimulate p53 signaling [22,23]. However, RASSF1A was shown to act by stimulating the intrinsic ubiquitination activity of MDM2, whereas we find that NORE1A is still active against a mutant form of MDM2 that cannot self ubiquitinate [21]. This may be explained by reports that RASSF1A acts to suppress β-TrCP activity [24], not stimulate it like NORE1A. Thus, the two RASSF family members may have different mechanisms of action that may be complementary in the regulation of p53.

NORE1A drives senescence by inducing pro-senescent post-translational modifications of both p53 [15] and Rb [25]. By suppressing MDM2, NORE1A could also promote the increase of both total p53 and Rb protein levels at the same time. To determine just how important MDM2 suppression is to NORE1A induced senescence, we over-expressed MDM2 in a NORE1A senescence assay. By overwhelming the MDM2 degradative system, we were able to severely inhibit the ability of NORE1A to induce senescence. Thus, NORE1A has a two-fold action on p53: enhanced acetylation to stimulate its pro-senescent transcriptional program [15] and stabilization of the total nuclear protein via suppressing MDM2. As we have also observed stabilization of Rb by NORE1A [25], it seems likely this effect may also involve MDM2. Further studies will be required to measure the relative effects on p53 and Rb stability. Epigenetic therapy strategies to restore NORE1A expression in tumor cells and recouple Ras to p53/Rb may have considerable potential for antagonizing Ras driven tumors.

## 4. Materials and Methods

### 4.1. Cell Culture

HEK-293, A549, and HepG2 cell lines all have wild type p53 and were obtained from the ATCC (Mannassas, VA, USA) and grown in DMEM supplemented with 10% Fetal Bovine Serum (Mediatech/Corning, Mannassas, VA, USA) and 1% Penicillin/Streptomycin (Corning, Mannassas, VA, USA). HEK-293 cells are well suited for transient transfections. A549 cells lack NORE1A and are therefore appropriate for NORE1A complementation studies. HepG2 cells retain NORE1A expression and are therefore useful for NORE1A knockdown studies. Cells were cultured in 5% CO_2_ at 37 °C. Transient transfections were performed using either jetPRIME® (Polyplus, Illkrich, France) or DNA-In^TM^ *A549* Transfection Reagent (Molecular Transfer, Gaithersburg, MD, USA).

### 4.2. Plasmids

Wild-type human MDM2 and mutant MDM2, unable to autoubiquitinate themselves (C464A), were obtained from Addgene (#20935 and #12086) [21]. pcDNA-HA-NORE1A, pEGFP-NORE1A, pEGFP-β-TrCP, pEGFP-C1-β-TrCPΔFBOX, and pmKate-2-C-H-Ras12V have been previously described [15,17,25].

### 4.3. Reagents, Western Blotting, and Antibodies

Total cell lysates were prepared by harvesting the cells in RIPA buffer (Sigma Aldrich Cat.# R0278, St. Louis, MO, USA) supplemented with a protease inhibitor cocktail (Sigma Aldrich Cat.# P8340) and sodium orthovanadate at a final concentration of 1 mM. Protein analysis on the cell lysates were performed using SDS-PAGE (NuPAGE 4%–12% Bis-Tris Gels, Life Technolgies, Waltham, MA, USA) transferred onto either nitrocellulose or PVDF. The membranes were subsequently probed with antibodies against HA (Covance Research Products, Greenfield, IN, USA), MDM2 (Santa Cruz Biotechnologies Cat.# SC-965, Dallas, TX, USA), GFP (Santa Cruz Biotechnologies Cat.# SC-9996), RFP (Evrogen Cat.# AB234, Moscow, Russia), β-TrCP (Cell Signaling Technolgies Cat.# 4394, Dallas, TX, USA), β-actin (Sigma Aldrich, St. Louis, MO, USA), and NORE1A (Rabbit poly-clonal described previously [15,25]). Western blots were developed using West Pico or West Femto Enhanced ECL detection systems (Thermo Fisher, Rockford, IL, USA) and exposed to blue chemiluminescent film. To inhibit the 26S proteasome, MG132 (Sigma Aldrich) was added to cell culture medium at a final concentration of 10 μM for 12 h.

### 4.4. Senescence Assay

A549 cells were transfected using DNA-In^TM^ *A549* and incubated for 72 h. The cells were then stained for β-galactosidase activity, a signature mark of cells undergoing oncogene induced senescence, using a kit from BioVision (BioVision, Milpitas, CA, USA). Cells were stained for 12 h and then quantitated using five random fields repeated over three assays per sample. Final quantitations are represented in percentage positive cells per field of view.

## 5. Conclusions

The Ras effector NORE1A acts, in part, by promoting the degradation of the negative regulator of p53 MDM2.
